# 

**Published:** 1894-01-06

**Authors:** 


### CONTENTS.

**Annus Medicus: 1893** - 209
**Modes of Administering Oxygen.** By Sir BENJAMIN WARD RICHARDSON, M.D., F.R.S. - 211
**Two Notable Doctors.**—Jean Martin Charcot—Sir Andrew Clark 212
**Studies in Applied Sociology**—I. Other People's Children - 213
**Art and Disease**—I. Possession or Hysteria - 214
**Annotations.**—A Medical Confessional—"Rights" in Hospitals—Bacillus Prodigiosus in the Pantry - 215
**Notes and News** - 216
**Medical Progress and Hospital Clinics**—
Spasm in the Diagnosis of Acute Meningitis in Children - 217
Case of Tubercular Peritonitis Complicated by Periosteal Abscess of the Thigh—Laparotomy—Recovery - 218
Injury as a Factor in the Production of New Growths of Bone - 219
MEDICAL PROGRESS.—Pleurisy - 220
Abdominal Surgery - 222
Ophthalmology. On Blepharitis, its Causes and Treatment - 223
**Current Medical Topics.**—Sanitary Deficiences and Puerperal Diseases - 224
**The Institutional Workshop**—
SOME SCOTCH ASYLUMS. By our Special Commissioner - 225
HOSPITAL FINANCE—I. Sources of Hospital Revenue - 226
HOSPITALS IN INDIA—I. The Bengal Presidency - 227
PRACTICAL DEPARTMENTS - 227
CONSTRUCTION NOTES - 228
**Scraps and Gleanings** - 228
**The Book World of Medicine and Science** - 229
**Medical Vacancies and Appointments** - vii

### EXTRA SUPPLEMENT.—THE NURSING MIRROR.

**News from the Nursing World.**—A Crimean Heroine—A Reasonable Request—Full and Empty Wards—Deserving of Support—A Good Worker—Valued Services—A Promised Hospital—Worthy of Imitation—Queen's Nurses at Inverness—Progress in Dublin—An Ideal Reception—Nursing in Ceylon—Her Own Wish—Only Three Pounds—Short Items cxxxi
**District Nursing.**—I. Introductory - cxxxiii
**A Few Remarks on Observation** - cxxxiv
**Our Examinations—Presentations** - cxxxiv
**Causerie from New England** - cxxxv
**"First Aid in Accident Cases." Programme of the Argentine Society** - cxxxvi
**Novelties for Nurses** - cxxxvi
**A Stroll Round the Wards** - cxxxvii
**Christmas Entertainments** - cxxxviii
**The Sussex County Hospital** - cxxxviii
**The Muses' Looking Glass.**—Woman Writers: Their Works and their Ways - cxxxix
**Queen Victoria's Institute for Nurses** - cxl
**The Book World for Women and Nurses** - cxl

